# Towards 100 Gbps over 100 km: System Design and Demonstration of E-Band Millimeter Wave Communication

**DOI:** 10.3390/s22239514

**Published:** 2022-12-05

**Authors:** Zeyuan Zhang, Xianbing Zou, Qun Li, Ning Wei

**Affiliations:** National Key Laboratory of Science and Technology on Communications, University of Electronic Science and Technology of China (UESTC), Chengdu 611731, China

**Keywords:** E-band millimeter wave, LoS MIMO, air-to-ground, long-range wireless communication system

## Abstract

Long-range E-band communication with fiber-equivalent speed is emerging extensively as a critical technology in the next-generation communication. This paper firstly reviews the relevant progress in recent research. A brief survey is presented on high-speed, long-range E-band communication systems and their relevant techniques that are essential to the link design, including antenna, power amplifier (PA), channel, and digital baseband processing. In the second part, we review our recent field trial of a long-range air-to-ground E-band link, which maintains steady transmission from a slow-moving helium balloon to the ground station with a vertical dimension of 20 km. The improvement directions and future research topics are then discussed.

## 1. Introduction

Along with the increasing bit rate in modern wireless access and backhaul transmission, the communication requirements in exceptional cases such as disaster recovery also underlie the growing demand for wireless systems with long range and high capacity. The exploitable bandwidth at high millimeter-wave frequencies has become an effective choice to meet the requirement. However, due to the solid atmospheric absorption, the communication range is severely limited for frequency bands around 60 GHz, 120 GHz, and 180 GHz, typically less than 20 km [1]. With 10 GHz available bandwidth right in the atmospheric window and robust transmission ability through dust and fog, the E-band (71–76 GHz, 81–86 GHz) communication systems are considered promising candidates to achieve 100 Gbps data rate at a range over 100 km, which is regarded as the technological objective in DARPA 100 Gb/s RF Backbone funding opportunity [2].

Given that signals in higher frequency have poor diffraction ability, line of sight (LoS) propagation in the millimeter wave is primarily considered under most validated scenarios. A terrestrial E-band link could be more easily impaired by shadow fading and earth curvature compared to other cases, such as aeronautics and air-to-ground communication. However, with the advance in antenna, high-power semiconductors, and advanced DSP techniques, various E-band communication systems with extensive coverage (tens of km) and high capacity (tens of Gbps) have been extensively emerging, both in terrestrial [3,4,5,6,7], and air-to-ground [8,9] scenario.

In this paper, we discuss in detail the system design and implementation of high-capacity, long-range E-band link. Thereafter, to further investigate the in-depth characteristic of the E-band air-to-ground channel and verify the platform’s performance in hand, a field transmission trial was also conducted.

The remainder of this paper is organized as follows. Section 2 introduces a brief survey of high-capacity, long-range E-band communication systems demonstrated in recent years and details the enabling technology essential to the link design. Section 3 reviews our field trial of an E-band air-to-ground link operated at 74 GHz from a slow-moving helium balloon to the ground station with a vertical dimension of 20 km. Section 4 discusses the encountered bottleneck and potential improvement in the future. Section 5 concludes the work.

## 2. Review on System Design

### 2.1. System-Level Demonstration

**Figure 1:**
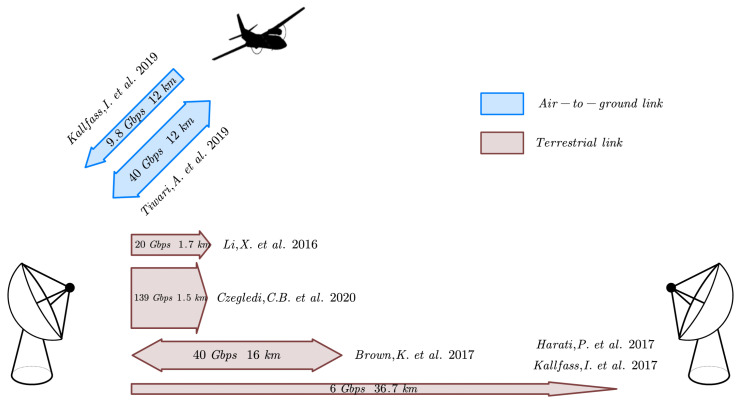
E-band long-range communication systems in the literature [3,4,5,6,7,8,9]. The two-way and one-way arrows represent the bidirectional and unidirectional link, respectively.

A brief survey of high-speed, long-range E-band wireless communication links in recent years is presented in this part. Notably, all works have reached a transmission rate over tens of Gbps at a considerable distance (see Figure 1).

The authors in [3] demonstrated a 1.7 km wireless link based on 2 × 2 multiple input multiple output (MIMO). Photonic millimeter wave generation was used to generate 85.5 GHz carrier frequency, after QPSK modulation and separated by a polarization beam splitter, the signal was amplified and fed into parallel 45 dBi Cassegrain antennas with H- and V-polarization, respectively, namely single polarized MIMO. The experimental result showed under the bit-error rate of 3.8 ×$10^{-3}$, the link can realize up to 20 Gbps data delivery at 1.7 km.

The terrestrial link proposed in [4,5] achieved up to 6 Gbps QPSK signal transmission at 36.7 km and 15 Gbps 8-QAM signal transmission over 15 km without applying multiplexing scheme. The power amplifiers were fabricated in GaAs/GaN mHEMT and provide about 80 dBm equivalent isotropic radiated power (EIRP) along with the 50 cm parabolic antenna. The Tx and Rx antennas were optically aligned to find the maximum receive power, and an overhead of around 15 dB is preserved for the atmospheric loss variations. The result showed that system bit error rate (BER) under rain and fog (4.6 ×$10^{-3}$) is inferior to that under clear sky (6.6 ×$10^{-8}$). Under the assumption of an ideal additive white Gaussian noise (AWGN) channel, the signal-to-noise ratio (SNR) generally has a 5 dB deterioration in comparison to the theoretical value derived from the link budget. However, the measured atmospheric loss showed good consistency with the ITU model, with a deviation of around 0.2 dB.

Furthermore, the link proposed in [6] provides an overall 80 Gbps bi-directional data rate over a range of 16 km. The transmit channel (71–76 GHz) and receive channel (81–86 GHz) are each separated into two subchannels with 2 GHz bandwidth, and the transmit waveform is duplexed with a corresponding receive waveform using high isolation duplexer. Thereafter, the polarization multiplexing was conducted by using an orthomode transducer (OMT). By doing so, four of the 2 GHz subchannel each sank by a BSM85100 with 10 Gbps data rate was multiplexed at the transceiver. E-band GaN power amplifiers with 1W saturated power were applied before each of the eight sub-channels combination to eliminate an inevitable 3 dB backoff. With 16-QAM Modulation, the link sustained an error-free (BER < 1 ×$10^{-8}$) bi-directional data rate of 44 Gbps at an equivalent range of 30 km. By applying 128-QAM, the system can provide an overall bi-directional bit rate of 76.2 Gbps (65 Gbps error-free) over 16 km.

To further increase the spectrum efficiency, Ericsson has presented a 139 Gbps LoS-MIMO link at 1.5 km operated at lower E-band with 2.5 GHz channel bandwidth [7]. Four 51 dBi parabolic dual-polarized antennas were deployed at both transmitter and receiver to set up an 8 × 8 LoS MIMO system. Limited by noise, the highest throughput was measured 139 Gbps with 256-QAM compared to the theoretical value of 144 Gbps (2.25 Gbaud × 8 bits/symbol × 8 streams), resulting in a spectrum efficiency of 55.6 bps/Hz. Moreover, the research has tested the robustness of both optimal and suboptimal deployments, i.e., offsetting the vertical separation between the receive antennas by 0.4 m and by 0.8 m.

In terms of air-to-ground transmission, the authors in [8] demonstrated a simplex communication link operating in 71–76 GHz between a microlight aircraft and a ground terminal. The aircraft flew with 1000 m height above the ground with a constant speed of 100 km/h, while the link can sustain a stable rate of 9.8 Gbps within 12 km. At RF frontend, GaN based solid-state power amplifier (SSPA) with 30 dBm output power was applied, and a 48.7 dBi steerable Cassegrain antenna at the ground terminal was aligned towards the aircraft based on GPS position information, whereas the transmit antenna with 39.7 dBi gain is packaged in a gimbal programmed to continuously point at the ground station to adaptively compensate the aircraft movement. Several modulation schemes were tested, the link BER is 7 ×$10^{-4}$ when 8 PSK modulation is applied in a 9.6 Gbps data rate.

In [9], the authors presented test results of an E-band link between a fixed ground station and a Cessna aircraft that flew at a top speed of 463 km/h in a 12 km air-to-ground slanted path, the link has achieved a bidirectional peak data rate of 40 Gbps. The transceiver aggregated 4 × 10 Gbps modulated carriers using a combination of frequency and polarization multiplexing. GaAs PAs with 33 dBm saturated output power was applied before signal was radiated by a 120 cm huge axially displaced ellipse (ADE) dual-reflector antenna with 59 dBi gain at ground terminal, and an 18 cm 42 dBi ADE at airborne. The highly sharp beamwidth was aligned by Global Position System (GPS)-based pointing, acquisition and tracking (PAT) process with accuracy better than 0.05°. Remarkably, the terminal provides the maximum bidirectional 40 Gbps data rates up to an altitude of 28 km and 10 Gbps up to 310 km.

### 2.2. E-Band Antenna

**Figure 2:**
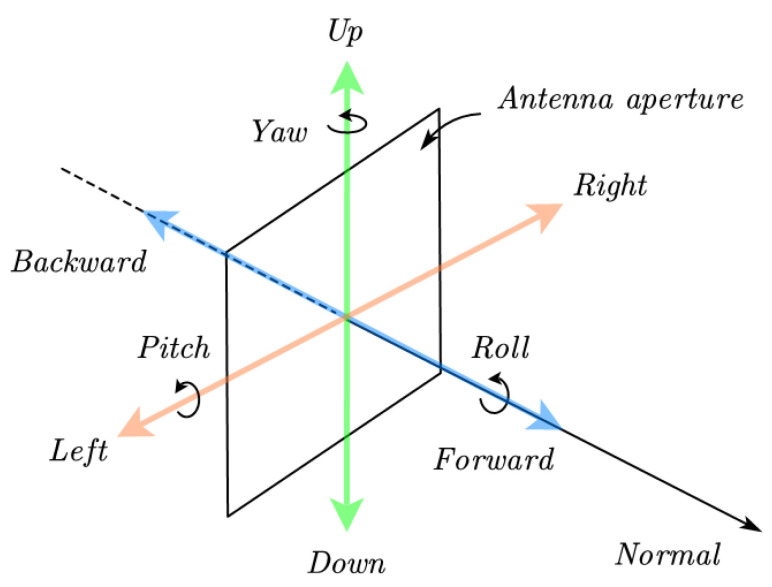
Antenna movement with degree of freedom 6.

The nonnegligible path attenuation and the atmospheric variation in long-range point-to-point communication links make signal power extremely precious for the systems. In E-band long-range communication, the essential requirement for antennas, except for the gain performance, is steerable beam, since antenna mast movement can increase the risk of outage by introducing additional attenuation (see Figure 2). This is further discussed in [10]. In this regard, the high-gain reflector/lens antennas with mechanical alignment and phased arrays seem to be the winning solution. Conventional Cassegrain antenna configuration suffers from secondary reflector blockage. Though the impairment can be compensated by shaping techniques and making a pointed vertex, it is still challenging to design an efficiently shaped Cassegrain reflector at higher frequency in the presence of diffraction effects [11]. ADE geometry can effectively address these problems with a displaced tilted ellipse along the axis, which eliminates the blockage due to the sub-reflector and the feed.

In [12], an E-band ADE reflector antenna with adjustable beamwidth was designed, reaching the highest directivity of 46.1 dBi. The adjustable beamwidth was achieved by mechanically changing the focus distance, which is also known as zoom antenna architecture. The half-power beam width (HPBW) of the proposed antenna can be expanded from 0.88° to 4.4° in the lower E-band and from 0.86° to 5.6° in the upper E-band. This variation in beamwidth is of great value in beam switching and the PAT process, allowing frontends steering with more flexibility. For a wider scan range, ref. [13] presented an E-band integrated lens antenna (ILA) fed by a 64-element beam-switching patch array integrated with diode switches on a multilayered liquid crystal polymer (LCP) substrate. The beam-switching range of ±4° × ±17° was achieved by changing the active antenna elements, which was controlled by an on-board switching network. The proposed ILA was applied later in an experimental E-band link to convey 700 Mbps data stream at a range of 55 m. However, the EIRP was constrained due to the significant mismatch of diode switches. The polarization multiplexing, enabled by polarized antennas, is commonly used in E-band antenna arrangement to improve the spectrum efficiency. Ref. [14] presented a high-gain ADE antenna fed by dual circular polarized horn antenna worked in W-band that reached directivity of 50 dBi. The cross-polarization discrimination (XPD) was higher than 28 dB for both left hand circular polarization (LHCP) and right hand circular polarization (RHCP). Another W-band dual-polarization reflector antenna work with RF sum-difference network was realized in [15]. The feeding network was composed of four OMTs and a coupling retracted structure. The sum of the opposite port is used to generate the sum beam of different polarization, while the difference in the opposite port is used to form the azimuth and pitch difference signal. By this means, the dual-polarization mono-pulse angle measurement can be conducted to identify the direction of arrival (DoA) of the incident wave, with a range of ±3°. The proposed antenna is measured to have 41 dBi gain, with remarkable cross-polarization level of 43 dB.

When it comes to phased arrays, according to the publication to date, the application still stays rare in E-band long-range transmission. This is partly brought by the manufactural challenge encountered by the E-band phase array, that is, with the operation wavelength around 4 mm, the overall antenna architecture design faces problems such as space constraints in circuit integration, concentrated heat dissipation, poor scalability, and strict manufacturing tolerances. In [16], a 16-element highly integrated W-band phased array, including the transceiver chipset manufactured in 0.18-μm SiGe BiCMOS technology was presented. The transceiver system integrates converters, phase lock loop (PLL), a 16-channel beamforming network, and integrated low noise amplifier (LNA) and PA for the receiver and transmitter, respectively. The transceiver integrated circuit (IC) was flip-chipped onto a low-cost organic printed circuit board (PCB) with two differently polarized antenna arrays fed by coupling apertures and stacked via. The measured EIRP of the transceiver is 34 dBm in each polarization which is enough to achieve 60 Gbps data transmission using 64-QAM at a distance of 1 m. The work was carried forward in [17] where the active phased array was extended into 384-Element. A maximum saturated EIRP of 60 dBm (1 kW) is measured at the boresight of the 256 transmit elements. The scalable architecture is preserved by incorporating the radio frequency integrated circuit (RFIC) and antennas into a PCB tile. The authors also established a wireless link operating at 90.7 GHz using the proposed phased array transceiver. The TX reached 52 dBm EIRP with an 8 dB backoff, 10 Gbps 16-QAM signal was demodulated with error vector magnitude (EVM) less than 8% over a distance of 265 m. In [18], a scalable 256-element E-Band active phased array was implemented on a 19-layer low temperature co-fired ceramic (LTCC) substrate, integrated with transceiver components fabricated in SiGe BiCMOS process. Due to the cost and system complexity constraints, hybrid beamforming was performed with 8 elements randomly tiled rectangular sub-arrays. The active phase array was tested in a 73 GHz link at 20 m with an EIRP of 35.5 dBm.

High-gain reflectors are mostly mechanically steered, which fails to accommodate the demand for fast networking and beam switching. Phased arrays can provide quick electronic scanning and flexible beam forming, but large phased arrays are more expensive and sometimes unpractical. The phased array fed reflector (PAFR), which received widespread attention in the radio astronomy community [19,20,21,22] may become an intermediate option to provide both considerable gain performance and a relatively limited electronic scanning region (usually 20°) and, of course, with lower cost. Generally, PAFR is composed of a reflector and a small phased array antenna on its focal plane, yet various forms of PAFR can be conducted by designing the reflector architectures (center/offset direct-fed, dual reflectors) and feed type (analog/digital phased arrays, switch matrix) [23].

The biggest challenge encountered by PAFR is the limitation of illumination area, that is, only limited array elements can be located in the area of maximum power density, which results in a limited EIRP level and the beam-scanning capability. As reported in [24], by increasing the axial displacement, a higher EIRP can be achieved in the PAFR antenna. For the mentioned 9 × 9 array fed reflector system working at 71 GHz, an axial displacement of 2.3 λ towards the reflector could provide an EIRP increase of 8 dB. However, this comes at the cost of a larger array size to meet the 80% efficiency criterion. Another challenge for conventional PAFR is the limited scanning range due to the beam deviation that occurs in the focal plane while performing wide-angle scanning. Ref. [25] introduced mathematical analysis to model the slightest deviation of the focused beam during wide-angle scanning when different kinds of reflectors were applied. The parabolic reflector is verified to have the largest illuminated region, which enables more elements to be involved in reflection, while during the wide-angle scanning process, a spherical reflector without axial displacements is found to be the best option for minimizing the lateral array size since it has the slightest deviation from the axis of revolution. In [26], a double shaped-reflector for focal plane array with wide FoV is presented. The limitation of illumination area and scan FoV in conventional PAFRs is addressed by introducing a secondary polynomial surface to gain extra control of the waves interacting with the array surface. By optimizing the geometric configurations, the proposed PAFR system can achieve scan angles up to 5° with 90% of array elements involved. For a scan angle up to 10°, the whole array is active. This comes at the cost of design complexity and sufficient size of the sub-reflector. However, the result showed a significant enhancement of 15 dB in EIRP compared to an equivalent conventional PAFR. On the extreme wide scanning scheme, The Lockheed Martin Corporation has proposed a novel architecture of PAFR which is called Wide Angle ESA Fed Reflector (WAEFR) [27]. An annular field of regard is created using ring-focus optics in dual reflectors. By custom beamforming, the system eliminated the need for an enormous feed array in wide-angle scan procedure. A zenith-facing electronically scanned array (ESA) working in conical scan mode and an ESA feed dual-reflector can cover the entire hemispherical FoV.

### 2.3. E-Band Power Amplifier

The PA plays a crucial role in the RF frontend of E-band long-range communication, for raising the transceiver EIRP could be the most straightforward solution to increase the SNR at the receiver. Consider a 73 GHz E-band link with 2 GHz bandwidth. Assume the required SNR at uncoded BER 1 × $10^{-6}$ (AWGN) is 12 dB, which is sufficient for QPSK demodulation. The noise figure of receiver NF is assumed to be 8 dB, and the antenna noise temperature $T_{a}$ = 290 K. So the system noise temperature of the receiver system can be obtained as

(1) $$T_{sys}=T_{a}+\left(10^{NF/10}-1\right)T_{0}=1830K$$

where $T_{0}=290$ K, according to

(2) $$SNR_{o}=\frac{S_{i}}{\left(kT_{SYS}B\right)}$$

We have the receiver input S_i_ = −61 dBm. where $k=1.38\times 10^{-23}$ J/K denotes Boltzmann constant and *B* denotes system bandwidth. For atmosphere attenuation of 0.4 dB/km, if the antenna gain is fixed at 40 dBi and a margin of 10 dB is preserved, a communication range of 20 km would require a PA with at least 33 dBm saturation power.

**Figure 3:**
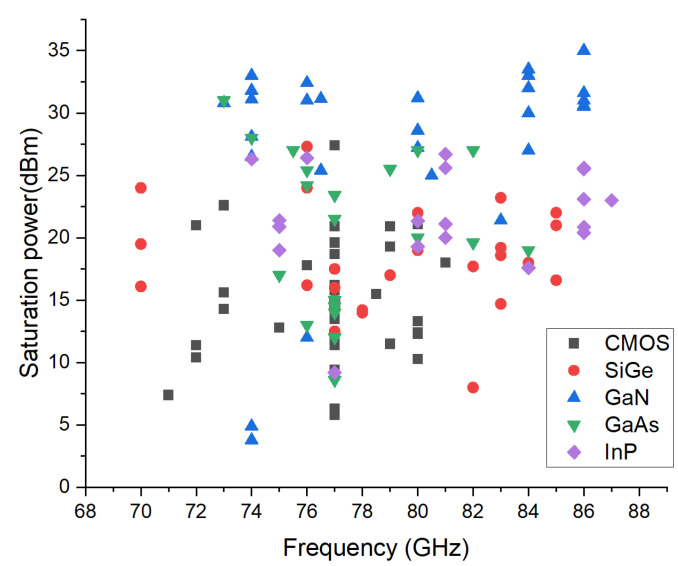
A Survey of the published E-band PAs, the plot shows saturated output versus frequency for different technologies, according to the data from [29].

A survey of published PAs in different technologies is shown in Figure 3. Though the PAs fabricated in Si-based technologies are superior in price and integrability, it can be observed that they are apparently limited by the output power. The InP-based technology excels by outstanding power performance above 100 GHz. The GaAs and GaN-based processes are considered more suitable for RF frontend in E-band communication. GaAs process can provide exceptional power density with great flexibility, which enables its use in different scenarios. However, when it comes to a watt level performance above 70 GHz, GaN seems to be the only promising candidate [28].

Under the funding of DARPA, the HRL lab has released a prototype monolithic microwave integrated circuit (MMIC) PA with 20 dB gain from 79–95 GHz using 40 nm GaN T3 process [30], the process can realize up to 200 GHz $f_{T}$, 400 GHz $f_{MAX}$ and a breakdown voltage up to 40 V, which provides PA with higher linearity, dynamic range, and RF survivability. The proposed MMIC PA achieves an output power of 1.3 W (31.3 dBm) at a remarkable power-added efficiency (PAE) of 27%. An 8.3 mm × 2.5 mm four-stage power amplifier circuit is proposed in [31] using OMMIC’s 60 nm GaN HEMT process, the saturated output power of the proposed PA is over 1.8 W (32 dBm), and the linear gain is more than 20 dB. It is worth noting that the remarkable flatness of ±0.2 dB is achieved to ease the burden of single carrier-frequency domain equalization (SC-FDE) systems. The PA operated in 71–76 GHz with PAE higher than 25%, which is also friendly to small size, weight, and power (SWaP) applications.

To further increase the output power, power combining using waveguide and low-loss transmission line media is widely applied to E-band PA to achieve 10 W+ output [32]. Ref [33] reported a W-band GaN power amplifier which could provide output power of 37 W (45.7 dBm) across the 75 to 100 GHz (28.6% relative bandwidth) and particularly above 45 W (46.5 dBm) in E-band (71–76 GHz, 81–86 GHz) application. This was accomplished by employing a radial off-chip waveguide combiner, which combines 12 broadband GaN MMIC chip modules. The power combining efficiency is 84.5% averaged across the band. In [34], a 100 W W-band GaN PA is also realized by low-loss split-waveguide power combining.

Higher output power can be achieved by on-die, waveguide, and spatial power combining approaches, but the power density remains the intrinsic factor. Ref. [35] demonstrated a wideband W-band GaN amplifier with an unique on-chip traveling-wave combiner design. The minimum outpower is 2 W over the 75–100 GHz bandwidth and reaches a peak of 3 W at 84 GHz, which can be further increased by employing a pulsed mode. A compact package size of 2.75 mm × 5.4 mm gives the amplifier power density of 2.1 W/mm^2^. In [36], a new 80 nm GaN HEMT process is used to fabricate a two-stage cascade unit, each has two transistors with the same gate periphery for high gain matching, which can provide 1.15 W (30.6 dBm) output power and 12.3% PAE at 86 GHz under continuous wave (CW) operation. The 2 × 1.8 mm^2^ MMIC reached the power density of 3.6 W/mm at W-band, which is the highest among the published research.

### 2.4. E-Band Channel Characteristic

#### 2.4.1. Atmospheric Influence

E-band radio transmission could be affected by several atmospheric factors. Among all the factors of regard, the rain rate is tested to have the most decisive influence on E-band radios except for free space loss. In [37], an experimental E-band link has been established to assess impairments of atmospheric attenuation. The link operated over 1.6 km with a constant symbol rate of 3.3 Gbaud at lower E-band. Both the QPSK and 16-QAM have been tested. According to the experiment result, the measured atmospheric attenuation shows good consistency with the ITU model, but the total propagation loss has differences in the absolute values, mainly in rainy conditions. These differences become greater for the cloudy days with cloud percentages over 60%. Moreover, the system shows about 1 dB performance improvement in colder temperatures and a 0.5 dB improvement during the night transmissions (under similar weather conditions). The attenuation caused by water droplets is investigated in [38] based on punctual weather parameters measured over two months. A rain attenuation model has been brought out with specific drop-size distribution (DSD), raindrop velocity, and scattering calculations. The authors also indicated a 0.1 dB negligible atmospheric attenuation difference between the upper cut-off frequency, i.e., 76 GHz, and the lower cut-off frequency, i.e., 71 GHz, at the lower E-band. Yet, the rain attenuation went through a difference of less than 1 dB, which leads to a 2% degradation in EVM at the exploited receiver. Ref. [39] showed the depolarization effect of a 72 GHz beacon signal in tropospheric weather events. The XPD degradation shows to be strongly correlated with rain rate, with variation around 20 dB. However, it behaves differently in the snow event, but it could be concluded that the precipitation type and precipitation rate both underlie the variation of XPD. For the air-to-ground propagation, the ITU (Rec. ITU-R P.840) indicates that cloud attenuation is related to the product of the attenuation coefficient $K_{l}$ ((dB/km)/(g/m^3^)) and liquid water density in the cloud, the former is given by

(3) $$K_{l}\left(f\right)=\frac{0.819f}{\varepsilon^{{}^{\prime \prime }}\left(1+{\left(\frac{2+\varepsilon^{{}^{\prime }}}{\varepsilon^{{}^{\prime \prime }}}\right)}^{2}\right)}$$

where *f* (GHz) is the frequency, $\varepsilon^{\prime }$ and $\varepsilon^{\prime \prime }$ are the real part and imaginary part of the dielectric permittivity of the water content according to the double-Debye model. Evaluation methods are also provided for gases (Rec. ITU-R P.676), scintillation (Rec. ITU-R P.618), and rain (Rec. ITU-R P.618). Though the influence of atmospheric behaviors in E-band millimeter-wave is still at the stage of observing and recording, which needs further investigation and modeling, the ITU model has been verified in various research with mild deviation and is considered an acceptable starting point.

#### 2.4.2. Channel Model

In [40,41], the experimental result showed that the variation in a 60 GHz LoS MIMO channel is relatively slow compared to the typical symbol durations in millimeter wave systems. Minor variations in the amplitude of the impulse responses could be found in a 1.25 GBaud transmission experiment. The mean value of the short-term phase variation is tested $1.3\times 10^{-5}\pi$/Symbol. Notably, it has been observed that the frequency selectivity due to the frontends has a dominant impact on the effective channel in comparison to the multipath effect.

It was once believed that the LoS MIMO channel failed to conduct multi-stream transmission for the lack of scatters on path, till it was proved that full-rank channel matrix could be achieved by spatially configuring the distribution of antennas, that is, the channel condition is strongly relevant to the deployment of MIMO antennas. For example, in reference [42], authors studied the fundamental characteristics of LoS MIMO channel with E-band uniform linear array (ULA) antennas at both receiver and transmitter ends. For the applications with limited physical size, the analysis showed that the maximum transmission distance to support spatial multiplexing with determined spatial streams and SNR is positively correlated with the product of the aperture size at receiver and transmitter arrays instead of numbers. In fact, the antenna arrangement that yields ideal spatial multiplexing can be determined not only for ULAs, but for a variety of array geometries [43,44,45]. Ref. [46] modeled the LoS MIMO channel capacity with respect to the arrays-of-subarrays (AOSAs) configuration and showed that quasi-optimal capacity could be reached as well as ULA with proper configuration and with less complexity in RF chains and precoders. The work in [47] considered a paradigmatic scenario, i.e., obliquely deployed ULA for strong LoS MIMO communications. By factorizing the channel gain matrix, the author proposed the orthogonal and rank-deficient arrangement with respect to the Rayleigh distance factor. Meanwhile, asymmetric LoS MIMO topologies with high spatial resolution on one side of the link, is proved capable of providing higher robustness for having full-rank channel matrices with distance and direction variation. This model is more practical for unfixed LoS MIMO communication.

Though the E-band backhaul channel for urban microcell and vehicle scenarios has been extensively investigated and modeled in [48,49,50], they are not suitable for long-range backhaul transmission over 10 km. If we consider a 2 × 2 MIMO with 10 km cover range at 73.5 GHz, according to the Rayleigh criterion

(4) $$R_{Ray}=\frac{N_{r}d_{t}d_{r}}{\lambda }$$

where $R_{Ray}$ denotes the Rayleigh distance, λ is the wavelength, $N_{r}$ is the number of antennas at the receiver, $d_{t}$ and $d_{r}$ denote the antenna spacing at the transmitter and the receiver, respectively. The product of antenna spacing at Tx and Rx would be at least 20 m^2^. For scenarios where antenna spacing is limited, e.g., an aircraft pod, such spacing will be unavailable, much less for a more extended range. Therefore, the deployment methodology for long-range transmission over the Rayleigh distance, as well as other in-depth characterization of E-band LoS MIMO channels still remains a challenging issue in the millimeter-wave realm and needs to be further studied. As a step in this direction, ref. [51] proposed a method of introducing specially designed phase variation, e.g., by adding dielectric material in the transmission path, to improve channel capacity. Different phase shifts can be introduced into different channels of LoS MIMO to reduce their correlation, which is further determined by the shapes and types of the dielectric medium. The extended LoS MIMO channel model with additional phase shift is showed to be capable of reducing the antenna aperture required by LoS MIMO system under the Rayleigh distance criterion or extending the Rayleigh distance at a specific antenna aperture. This could be enlightening for an E-band long-range air-to-ground link modeling in the presence of a single-layer cloud. Conventional cloud modeling, such as Cloud Sense Simulation (CSS) and modified Fractal Brownian Motion (FBM), can give meteorologically and visually feasible models of clouds with great complexity and show poor adaption to the air-to-ground LoS MIMO scenario. We therefore proposed a cloud modeling method to analyze the additional phase variation obtained by electromagnetic waves in the single-layer cloud with randomly distributed ice and moisture component, which shows good consistency to the result in the practical measurement which is reviewed in Section 3. The model, as well as a preliminary evaluation of the influence of cloud in air-to-ground LoS MIMO channel, is presented in [52].

### 2.5. Digital Baseband

#### 2.5.1. Modulation Scheme

High-order modulations are preferred in E-band high-speed transmission but are usually limited by their implementation complexity and the high sensitivity to phase noise and IQ imbalance. Thus various automatic modulation classification (AMC) methods have been brought out to identify the modulation scheme adaptively based on features extraction [53,54]. On the other hand, constellation shaping has emerged as an attractive technique to improve the SNR of M-QAM systems in linear regimes [55]. By probabilistically and geometrically arranging the signal mapping, the modulation performance can be customized.

In [56], probabilistic amplitude shaping combined with orthogonal circulant matrix transform (OCT) precoding was proposed and experimentally demonstrated in a 64-QAM orthogonal frequency division multiplexing (OFDM) based W-band radio over fiber (RoF) system. Authors in [57] proposed a new probabilistic shaping scheme for 16 and 64 amplitude phase shift keying (APSK). The source was converted into a stream of Rayleigh distributed symbols in the first quadrant by distribution matcher (DM). Thereafter, a systematic low-density parity check (LDPC) encoder was used to generate 2 parity bits per symbol to determine the quadrant for the transmitted symbol. According to the simulation, the proposed PS-APSK modulation could provide 0.9 to 2.1 dB lower peak to average power ratio (PAPR) and higher SNR compared to PS-QAM at the same average power. In terms of geometric shaping, ref. [58] proposed an optimized 4-4-4-4-N and C-32QAM-N modulation scheme for the millimeter-wave receivers. The proposed circular 16-QAM (4-4-4-4-N) is constructed by minimizing the average energy at the determined minimum Euclidean distance (MED) and then tuning the distance between the concentric rings to minimize PAPR. Better tolerance in critical phase can be obtained using the proposed scheme compared to the existing works reported in the literature. The result is further verified in an experimental 60 GHz millimeter wave transmission link. For low-PAPR modulation in energy-efficient applications, research [59] proposed constrained phase-shift keying (CPSK) in the discrete Fourier transformation (DFT) spread OFDM system. Additional constraints are applied to conventional PSK, where the symbol transitions are limited to some of the closest neighbors of the current symbol. The bit-to-symbol mapping works in even and odd time mode with the presence of a constellation rotation to further reduce the PAPR. The authors later evaluated the modulation by applying the PA model defined for IEEE 802.11ad in [60]. While attaining a similar EVM performance, the CPSK has a 1-2dB cut down in PAPR and acts more positively in maximizing the PA output power compared to the conventional QPSK and π/4-QPSK with no extra complexity introduced to the modulator, which seems appealing in the energy-constrained scenario.

#### 2.5.2. LoS MIMO Equalization

**Figure 4:**
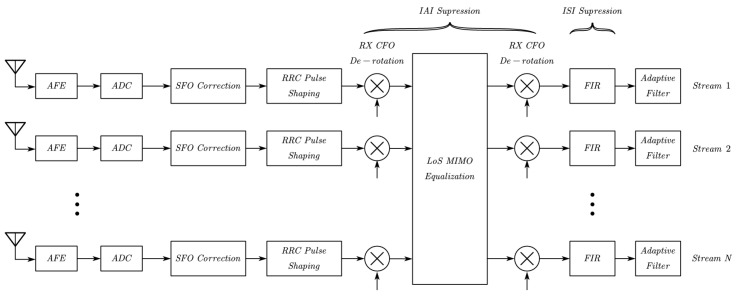
Block diagram of the proposed ’STIAI+PISIE’ structure in [62].

Conventional equalization methods, i.e., zero-forcing (ZF) and minimum-mean-square-error (MMSE), are commonly used in MIMO systems. With developement in machine learning, multiple artificial intelligence (AI) strategies are also deployed in equalization to achieve faster execution at the signal processing level with highly-parallel computing [61]. Considering the characteristic of LoS MIMO systems, plenty of research works have been brought out to improve the equalization performance. Authors in [62] proposed a temporal efficient frame structure for stationary LoS MIMO systems with frequency-selective and time-varying components. Orthogonal training sequences were sent as preambles and midambles to estimate the channel state information (CSI) and carrier frequency offset (CFO), which are further introduced in IAI suppression. According to the decomposition of the effective channel matrix, the IAI was suppressed successively by RX CFO de-rotation, MIMO equalization and TX CFO de-rotation with linear complexity. The frequency-selectivity is found rather similar for different streams due to the usage of the similar analog frontend (AFE), so the authors suppressed ISI in parallel by exploiting a fixed MMSE-finite impulse response (FIR) filter per stream, as shown in Figure 4. The proposed low-complexity algorithm was verified in a 60 GHz hardware-in-the-loop 2 × 2 LoS MIMO demonstrator and outperformed the joint equalization using the general version decision-directed least-mean-square (DD-LMS) algorithm.

A sequential channel equalization was proposed in [63] to reduce the complexity and improve energy efficiency. The LoS MIMO matrix was factorized into three parts, with the diagonal matrices outside and the middle one dominated by an inverse-DFT matrix considering arrangement displacements along the transmission direction. Equalization was performed sequentially in reverse order with respect to the channel factorization, where DFT operations can exploit the benefits of the butterfly designs in fast Fourier transform (FFT) to reduce the complexity. The overall complexity of the proposed equalization is almost linearly with respect to the multiplexing streams. A 60 GHz 2 × 2 LoS MIMO link was used to verify the proposed equalization, which has approximate performance in comparison to the ZF algorithm with lower complexity. In [64], a novel adaptive space-time equalization for the frequency-selective channel has been proposed to handle geometric misalignment for LoS MIMO, which could introduce additional ISI and cross-stream interference (CSI). A Spatial oversampling was conducted by increasing the number of receivers. Adaptive windows in the time domain were employed at each receiver to capture a certain energy level of the currently desired symbol affected by both ISI and CSI to construct a signal space against the interference. The linear minimum mean squared error (LMMSE) equalizer with respect to the temporally and spatially extended channel matrix is then calculated separately for each multiplexed stream to conduct a space-time equalization. The BER performance is simulated better as the spatial oversampling factor and the window length increase, which can be attained adaptively using a greedy strategy. However, the proposed equalization requires a dedicated RF chain for each antenna in the presence of spatial oversampling, which causes increase in cost.

#### 2.5.3. Impairment Compensation

- Phase Noise

The phase noise could cause a series of problems, such as constellation rotation, adjacent channel interference (ACI), and inter-carrier interference (ICI) in E-band transceivers if not compensated. The IEEE 802.11 ad [65] recommends the single-pole/zero model to capture the power spectral density (PSD) with

(5) $$S\left(f\right)=K_{0}\frac{1+{\left(f/f_{z}\right)}^{2}}{1+{\left(f/f_{p}\right)}^{2}}$$

where $K_{0}$ denotes the PN level and $f_{p}$ and $f_{z}$ are pole and zero frequencies, respectively. A detailed performance evaluation for LoS MIMO systems with the presence of phase noise is performed in [66]. In order to mitigate the impairment caused by phase noise in the millimeter-wave band, a compensation method based on Golay sequence was introduced in [67]. Authors in [68] proposed an iterative decision-aided phase noise compensation scheme for millimeter systems compliant with IEEE 802.11ad standard. The received data block is divided into subblocks where the phase noise is considered stationary. After the equalization, an initial decision was made by considering the phase noise in the whole data block as constant, and by using the former demodulated data, a maximum likelihood (ML) estimation for the phase noise is conducted in each subblock. As the iteration continues, the data block is divided into more pieces, and better data decisions can then be obtained. The proposed algorithm has low complexity and performs better when the iteration number goes greater compared to the conventional decision-directed compensation schemes in [69]. Ref. [70] described a simple phase noise compensation scheme for wideband processing in SC-FDE systems considering the multi-pole/zero PN model, where the recovered unique word (UW) samples are linearly combined to average the error before estimating the phase noise in each data block. The algorithm has the merit of being delay-free and is easy to implement in the physical layer since no iteration is required. Research [71] has developed a low-complexity phase noise suppression for millimeter-wave OFDM systems. In this work, the phase noise is modeled by a specific order of dominant components in the frequency domain. Then integral estimation of the PN-affected channel is conducted by an LMMSE estimator, where a look-up table based on uniformly quantized SNR is used to eliminate the matrix multiplication and inversion. The proposed method has low hardware complexity and has been verified on a low-cost very large scale integrated (VLSI) circuites architecture with 258 K gate count and power consumption of 19.3 mW at 250 MHz clock rate.

- IQ Imbalance

The IQ imbalance is usually classified into frequency-independent (FI) imbalance and frequency-dependent (FD) imbalance. The former is introduced by the non-ideality oscillators and shows stationary feature over the bandwidth, while the latter is caused by the frequency-selective components, e.g., matched filters, analog to digital converter (ADCs) and varies over the bandwidth.

**Figure 5:**
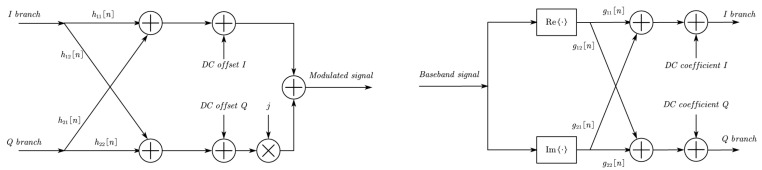
The baseband-equivalent L-tap model of the I/Q modulator (**left**) and I/Q compensator (**right**).

A training-based compensation scheme of IQ Imbalance for systems compliant with IEEE 802.11ad standard is researched in [72]. In the SC-FDE scene, the received baseband signal was modeled with respect to the imbalance and channel information. A likelihood function can be derived with the help of the training sequence. Then the IQ imbalance and channel parameters can be estimated jointly and separately by iteratively maximizing the likelihood function. Finally, compensation and equalization are conducted in the frequency domain. The IQ imbalance on the transmitter side was especially considered in [73] by applying the same model, and the simulation shows that a single iteration is sufficient to approach the benchmark of the ideal case without TX IQ imbalance. Ref. [74] indicated a special frequency-dependent IQ imbalance introduced by AD/DAs, i.e., the in-phase and quadrature timing mismatch (IQTM), which adversely impacts MIMO OFDM-based millimeter-wave system. Various RF distortions, including CFO, PN, sampling frequency offset (SFO), and sampling time offset (STO), were considered in the modeling. The author also proposed a pilot-based joint estimation metric for TX and RX IQTM and the corresponding compensation scheme. Authors in [75] proposed a method to compensate for the frequency-dependent IQ imbalance in wideband direct-conversion millimeter wave transmitters. A transmitter-observation receiver (TOR) design is implemented where a branch of the signal is down-converted to the intermediate frequency (IF) and under-sampled by an ADC after the PA amplification to fit the training algorithm of digital predistortion and IQ compensation. With this TOR feedback procedure, nonideal parameters of the IQ modulator can be estimated by recursive least-squares (RLS) algorithm and compensated by the inverse L-tap structure, as shown in Figure 5. The presented method was experimentally verified in conjunction with the digital predistortion (DPD) process, the compensation scheme shows about 4% reduction in the root normalized mean-square error (RNMSE) of the IQ modulator output signal. The digital predistortion training can be conducted using the same TOR architecture with no restrictions on the IF setting.

- PA Distortion

With the application of high-order modulation, the PA linearity grows into one of the major concerns in the E-band long-range backhaul. Previous architectures such as Doherty and envelope-tracking (ET) have been proved to introduce additional distortion into the signal [76]. In this regard, DPD is widely applied in the digital baseband to maximize the output power and energy efficiency.

Ref. [77] first demonstrated DPD improvement in PA efficiency and in-band distortion at E-band with 2 GHz signal bandwidth. A commercial E-band GaAs PA was deployed in a 1.6 Gbaud direct learning loop, the corresponding digital predistorter was implemented by a simple 5th-order memoryless polynomial function. To find the optimal coefficients, the authors started with a behavior modeling technique and optimized the result by exhaustive searching and The Powell’s conjugate direction optimization method. The latter is proved to reach the optimal sets within tens of seconds. The proposed DPD can generally increase the output power by 2–3 dB at a specific EVM, which brings the utmost 100% efficiency improvement for a linear PA operation. In [78], an open-loop predistortion was implemented in an E-band communication link to improve signal quality and adjacent channel power (ACP). The authors demonstrated the performance improvement under different channel bandwidths up to 1 GHz from 71 to 76 GHz. By introducing the dynamic deviation reduction (DDR) technique, the Volterra series is simplified and exploited in the DPD. In the performed experimental loop, 3.8 dB of EVM improvement can be observed around the 1 dB compression point after applying the DPD algorithm. The authors also noted that the EVM improvement increases for higher bandwidths, whereas the ACP improvement decreases. In [79], a novel DPD technique that can simultaneously eliminate the cross-polarization interference (XPI) of dual linearly-polarized (DLP) antenna and distortion of PAs in millimeter-wave transmitters was proposed. Firstly, the signal affected by both effects is collected by over-the-air (OTA) tests, then coefficients of both compensation modules can be extracted from the collected signals by applying an iterative decomposition algorithm. Thereafter, predistortion is implemented in the cascaded XPI cancellation and nonlinear compensation. According to the dual-polarized transmission experiment, the proposed DPD had significant improvement in both normalized mean square error (NMSE) and adjacent channel power ratio (ACPR) compared to the memory polynomial based method without XPI compensation.

## 3. Demonstration of An Experimental Air-to-Ground E-Band Link

To acquire a more profound knowledge of the atmospheric transmission characteristic and multipath effect for E-band long-range air-to-ground channels, we conducted a test campaign at Qinghai, China, located at coordinates 37.7° N, 95.3° E, on 27 September 2021.

### 3.1. System Overview

**Figure 6:**
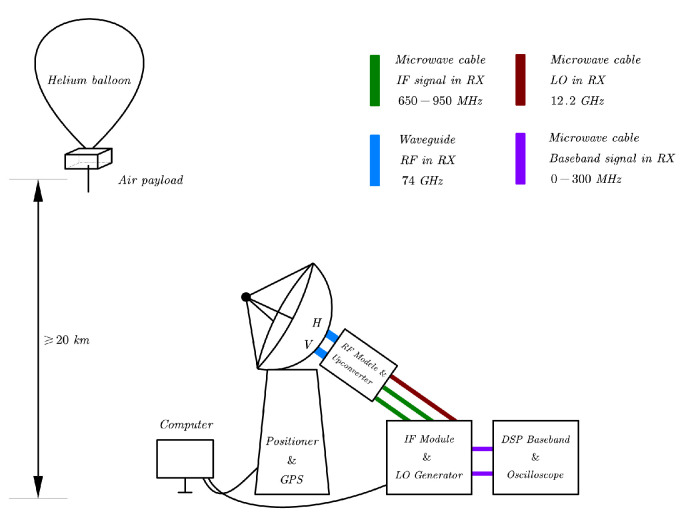
Schematic diagram of the experiment system.

As shown in Figure 6, a simplex air-to-ground transmission is implemented. The transmitter was placed in a slow-moving helium balloon platform with a peak rate of 10 m/s, while the receiver was previously arranged on the ground during the flight path. After the ascentstage, the helium balloon platform maintained the level flight at an altitude above 23 km, with the ambient temperature ranging between 13° and 35° and air pressures between 2.3 and 5.1 kPa. Our transmission experiment was conducted in the predetermined flight path where the cloud gradually receded.

#### 3.1.1. Transmitter and Air Payload

**Figure 7:**
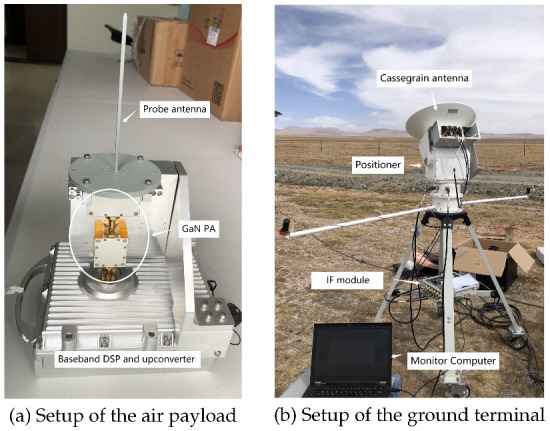
Implementation of the transmitter over the air and receiver on the ground terminal.

The transmitter hardware consists of a probe antenna, a solid-state power amplifier, an upconverter, and baseband field programmable gate array (FPGA) electronics. The beam width of the probe antenna is 60°, which can provide a reachable circular area with a diameter of 23 km on the ground. A GaN power amplifier is adopted with an available output power of 39 dBm at an average PAE of 16%. The FPGA is programmed to continually deliver the data frames previously stored in the ROM with a baud rate of 250 MHz. The baseband signal is first modulated to 800 MHz IF and then upconverted to 74 GHz. The whole transmitter has an EIRP of 44 dBm and is fixed securely in the pod of the helium balloon. Figure 7a shows the setup of the air payload, the upconverter, the local oscillator (LO) generator, and all digital baseband electronics are packaged in the cabinet.

#### 3.1.2. Receiver and Ground Terminal

**Figure 8:**
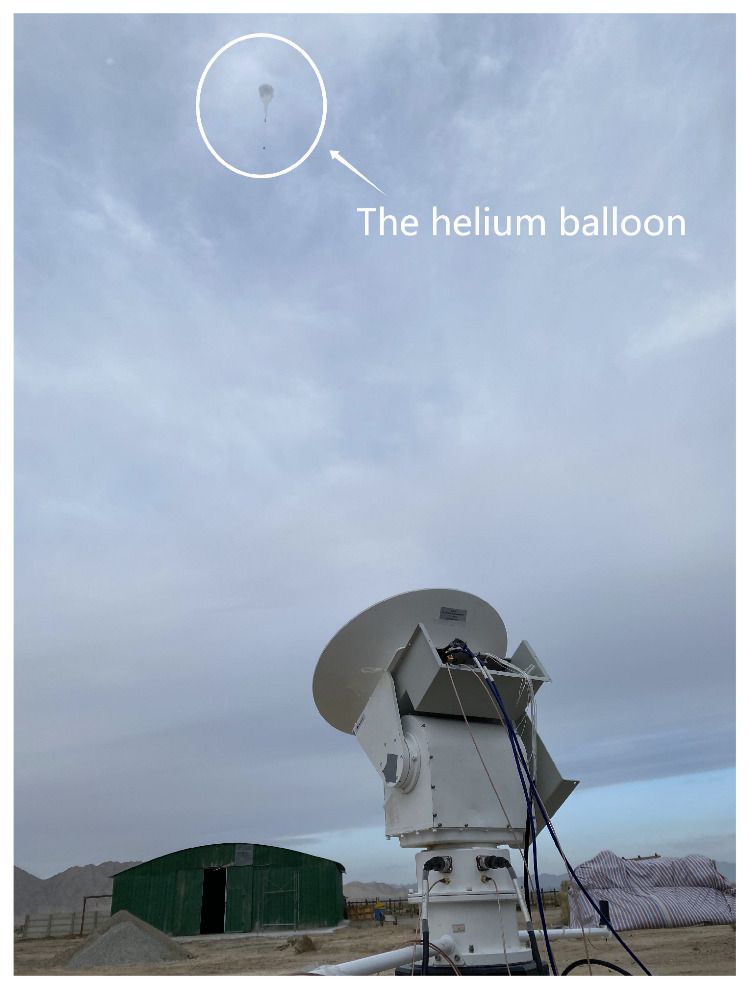
The steerable Cassegrain antenna tracks the air balloon in the ascent stage.

As is shown in Figure 7b, the implementation of the receiver consists of a dual-polarized steerable Cassegrain antenna, an IF module, and digital baseband electronics. The 35 cm Cassegrain antenna can provide 44 dBi gain, resulting in a highly directional beam with HPBW of 0.7°. The antenna positioner is functioned by a gimbal motor which is programmed to continually tracks the balloon with the help of GPS information, Figure 8 shows the tracking procedure in the ascent stage of the balloon. After the antenna, a low-noise receiver down-converts the received signal to 800 MHz IF using the ×6 multiplication frequency of the 12.2 GHz LO signal. After being converted to the analog baseband, the signal is digitized and further processed in the digital domain. The processed data are packetized and sent to the software and finally displayed on the monitor. Table 1 illustrates the key parameters of the system.

The auxiliary equipment at receiver side consists of an AD converter, a disk array and a monitor computer. The 1 Gsps sampling AD is connected directly to the modem for a consecutive data acquisition about 20 min. Sampled signals are then stored into the disk array for future analysis, the computer is used to monitor the beam alignment situation and communication performance of the received signal, including spectrum, constellation and throughput.

### 3.2. Experimental Result and Discussion

**Figure 9:**
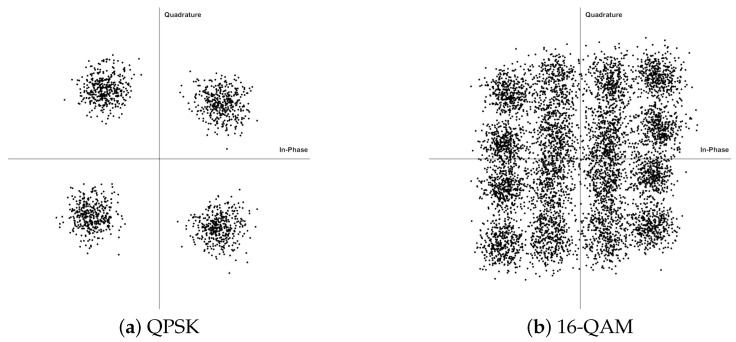
Constellation diagram of the received siganl and LoS distance of 20 km.

**Figure 10:**
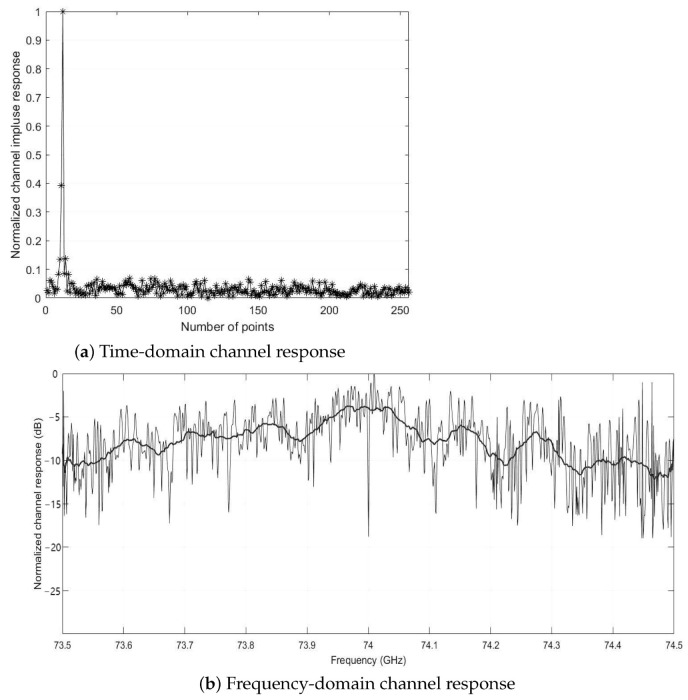
Estimated channel response at LoS distance of 20 km.

The measurement is conducted under a clear sky condition after the cloud receded, which results in an average atmospheric attenuation of 0.12 dB/km. Real-time DSP including polarization selection combining, synchronization, CFO, and fractional frequency domain equalization is finished in the baseband FPGA with 24.8 W power consumption. Off-line data are also preserved for the future modification in DSP algorithms and the air-to-ground channel analysis, which is further discussed in [52]. Figure 9 shows the 250 MBaud signal quality at LoS distance of 20 km. The transmission remained stable for more than 30 min during the level flight stage. We integrate the Golay sequences into the data frames to estimate the long-range air-to-ground channel, and one of the normalized channel responses averaged over 3 data frames (0.73 ms) at 20 km LoS distance is shown in Figure 10. Due to the conservative hardware setup, there is still room for improvement in the future considering the implementation of MIMO structure.

## 4. Open Issues and Future Research Directions

This section discusses a few open research problems that have not been fully studied in the literature and require further research attention.

### 4.1. Multi-Stream Signal Processing of LoS MIMO Channel over Rayleigh Distance

In a conventional LoS MIMO scenario, the multiplexing gain tends to decrease for longer distances, which limits the transmission rate with a restricted power level. Approaches to reach beyond the Rayleigh distance are still one of the open issues in the long-range wireless backhaul. In this regard, the multiplexing gain variation caused by the heterogeneous medium in the transmission environment still needs to be further studied, since it is possible to extend the coverage by introducing different phase variations in the link [51]. Under this assumption, the efficient MIMO multiplexing scheme, including the optimal antenna deployment for non-uniform antenna array and combination with dual/multi-polarization, is likely to boost the performance of E-band high-speed backhauls.

### 4.2. Atmospheric Influence and Adaptive Link Management

The attenuation effect due to precipitation has been investigated in multiple research. However, the in-depth characteristic of the atmospheric influence, e.g., the phase imbalance and XPD degradation due to different weather conditions [39], as well as the tropospheric turbulence and scintillation effect in E-band long-range channel still need to be further studied. To date the progress is still in preliminary observation. Due to the link variation, an adaptive link management scheme, i.e., the adaptive combination of multiple modulation formats and coding, shall be further considered for a robust and reliable transmission.

### 4.3. High Accuracy Long-Range Beam Alignment

Due to the highly directional beam in E-band long-range communication, systems are susceptible to beam mismatch. Despite the mechanically assisted reflectors are widely used, dull beam switching and troublesome tracking procedure make it unsuitable for dynamic link and multi-user applications. In this regard, the PAFR, which can offer fast inertia-free scanning with considerable gain and modest cost, might be a feasible solution for future implementation. Resolution to the common defect of PAFR, such as restricted illuminated region and wide scanning deviation, is of interest in recent investigation. On the other hand, the research on joint control algorithms of mechanical and electrical beam steering and fast beam detection method is also essential to dynamic networking.

### 4.4. High-Speed Multi-Stream Detection and Decoding

Due to the extremely high throughput in future E-band communication, a feasible VLSI implementation is necessary to handle multi-stream signal processing on a large computational scale with up to 100 Gbps. Stochastic computing-based FIR filters and FFT converters [80,81] are considered hopeful to further improve the area and energy efficiency. On the other hand, emerging analog computing brings more significant advantages in speed and power consumption, e.g., [82] have introduced 16 and 64-point analog fast Fourier transform (AFFT) while consuming tens of mW. Resistive processing units can compute matrix-vector multiplication in the analog domain in O(1) time, which gives a considerable boost in signal processing as well as AI and shows the potential to even entirely eliminate the intermediate AD/DA process [83,84].

## 5. Conclusions

In this paper, we introduce a brief survey of the prevailing long-range, high-capacity E-band communication systems and their enabling technology, i.e., antenna, power amplifier, channel, multiplexing, and digital baseband processing. We experimentally demonstrate an E-band long-range air-to-ground transmission link operated at 74 GHz from a helium balloon to the ground station with a vertical dimension of 20 km, which could be a very start towards the 100 km-100 Gbps objective. In this experimental transmission, we preliminarily verify the system feasibility and channel performance above the troposphere with a conservative hardware setup. Finally, We discuss the open research issues and challenges that deserve future research attention. It is reasonable to be positive that E-band high-speed long haul will thrive in the near future.

## Figures and Tables

**Table 1:** Experimental air-to-ground link parameters.

Parameter	Specification
Center frequency	74 GHz
Symbol rate	250 MBaud
Roll-off factor	0.2
Modulation scheme	BPSK, QPSK, 16-QAM
**Transmitter**	
PA output	38 dBm
Antenna gain	6 dBi
EIRP	44 dBm
**Receiver**	
Antenna gain	44 dBi
Antenna polarization	Vertical and horizontal
Antenna cross-polarization isolation	≥30 dB
Thermal noise	−89 dBm
Noise figure	7 dB

## Data Availability

Not applicable.

## Abbreviations

The following abbreviations are used in this manuscript:

PA	Power amplifier
DARPA	Defense Advanced Research Projects Agency
RF	Radio frequency
LoS	Line of sight
DSP	Digital signal processing
MIMO	Multiple input multiple output
QPSK	Quadrature Phase Shift Keying
QAM	Quadrature amplitude modulation
EIRP	equivalent isotropic radiated power
BER	Bit error rate
AWGN	Additive white Gaussian noise
SNR	Signal to noise ratio
OMT	Orthomode transducer
SSPA	Solid state power amplifier
ADE	Axially displaced ellipse
PAT	Pointing, acquisition and tracking
FoV	Field of view
HPBW	Half power beam width
ILA	Integrated lens antenna
LCP	Liquid crystal polymer
XPD	Cross-polarization discrimination
LHCP	Left hand circular polarization
RHCP	Right hand circular polarization
DoA	Direction of arrival
PLL	Phase lock loop
LNA	Low noise amplifier
IC	Integrated circuit
PCB	Printed circuit board
RFIC	Radio frequency integrated circuit
EVM	Error vector magnitude
LTCC	Low temperature co-fired ceramic
PAFR	Phased array fed reflector
WAEFR	Wide Angle ESA Fed Reflector
ESA	Electronically scanned array
PAE	Power-added efficiency
MMIC	Monolithic microwave integrated circuit
SC-FDE	Single carrier-frequency domain equalization
SWaP	Small size, weight and power
CW	Continuous Wave
ITU	International Telecommunication Union
DSD	Drop-size distribution
ULA	Uniform linear array
AOSAs	Arrays-of-subarrays
CSS	Cloud Sense Simulation
FBM	Fractal Brownian Motion
AMC	Automatic modulation classification
OCT	Orthogonal circulant matrix transform
OFDM	Orthogonal frequency division multiplexing
RoF	Radio over fiber
APSK	Amplitude phase shift keying
DM	Distribution matcher
LDPC	Low density parity check
PAPR	Peak to average power ratio
MED	Minimum Euclidean distance
CPSK	Constrained phase-shift keying
DFT	Discrete Fourier transformation
ZF	Zero-forcing
MMSE	Minimum-mean-square-error
AI	Artificial intelligence
CSI	Channel state information
CFO	Carrier frequency offset
FIR	Finite impulse response
DD-LMS	Decision-directed least-mean-square
FFT	Fast Fourier transform
CSI	Cross-stream interference
LMMSE	Linear minimum mean squared error
PSD	Power spectral density
ML	Maximum likelihood
UW	Unique word
VLSI	Very large scale integrated circuites
AD	Analog to digital
DA	Digital to analog
ADC	Analog to digital converter
FI	Frequency-independent
FD	Frequency-dependent
IQTM	In-phase and quadrature timing mismatch
SFO	Sampling frequency offset
STO	Sampling time offset
TOR	Transmitter-observation receiver
RLS	Recursive least-squares
RNMSE	Root normalized mean-square error
IF	Intermediate frequency
ET	Envelope-tracking
DPD	Digital predistortion
ACP	Adjacent channel power
DDR	Dynamic deviation reduction
XPI	Cross-polarization interference
DLP	Dual linearly-polarized
OTA	Over-the-air
NMSE	Normalized mean square error
ACPR	Adjacent channel power ratio
FPGA	Field programmable gate array
LO	Local oscillator
GPS	Global Position System
Gsps	Giga sample per second
BPSK	Binary phase shift keying
AFFT	Analog fast Fourier transform
